# A Novel Peptide Antibiotic, Pro10-1D, Designed from Insect Defensin Shows Antibacterial and Anti-Inflammatory Activities in Sepsis Models

**DOI:** 10.3390/ijms21176216

**Published:** 2020-08-27

**Authors:** Manigandan Krishnan, Joonhyeok Choi, Ahjin Jang, Yangmee Kim

**Affiliations:** Department of Bioscience and Biotechnology, Konkuk University, Seoul 05029, Korea; biomani1@konkuk.ac.kr (M.K.); jun9688@konkuk.ac.kr (J.C.); ajin931017@konkuk.ac.kr (A.J.)

**Keywords:** antimicrobial peptide, gram-negative infection, peptide antibiotics, biofilm, sepsis

## Abstract

Owing to the challenges faced by conventional therapeutics, novel peptide antibiotics against multidrug-resistant (MDR) gram-negative bacteria need to be urgently developed. We had previously designed Pro9-3 and Pro9-3D from the defensin of beetle *Protaetia brevitarsis*; they showed high antimicrobial activity with cytotoxicity. Here, we aimed to develop peptide antibiotics with bacterial cell selectivity and potent antibacterial activity against gram-negative bacteria. We designed 10-meric peptides with increased cationicity by adding Arg to the N-terminus of Pro9-3 (Pro10-1) and its D-enantiomeric alteration (Pro10-1D). Among all tested peptides, the newly designed Pro10-1D showed the strongest antibacterial activity against *Escherichia coli*, *Acinetobacter baumannii*, and MDR strains with resistance against protease digestion. Pro10-1D can act as a novel potent peptide antibiotic owing to its outstanding inhibitory activities against bacterial film formation with high bacterial cell selectivity. Dye leakage and scanning electron microscopy revealed that Pro10-1D targets the bacterial membrane. Pro10-1D inhibited inflammation via Toll Like Receptor 4 (TLR4)/Nuclear factor-κB (NF-κB) signaling pathways in lipopolysaccharide (LPS)-stimulated RAW264.7 cells. Furthermore, Pro10-1D ameliorated multiple-organ damage and attenuated systemic infection-associated inflammation in an *E. coli* K1-induced sepsis mouse model. Overall, our results suggest that Pro10-1D can potentially serve as a novel peptide antibiotic for the treatment of gram-negative sepsis.

## 1. Introduction

Overwhelming pathogenic stimuli can lead to the progression of host immune response to systemic inflammatory response syndrome (SIRS), increasing the severity of infections and ultimately inducing pathological processes of sepsis. Sepsis is a multifaceted syndrome caused by an aberrated immune response to bacterial infections, thereby culminating in excessive inflammation, organ dysfunction, and even death [1,2]. According to a nationwide cohort study, the incidence of sepsis cases in Korea has increased, with approximately 233.6 per 100,000 people affected and a 22.6% mortality in 2016 [3]. Especially, infections by gram-negative bacteria are an urgent problem because the outer cell membrane of bacteria consists of lipopolysaccharide (LPS), which not only triggers aberrant cascades of the inflammatory response but also hinders development of new antibiotics against resistant infections [4,5]. Several studies have revealed that the disproportionate activation of toll-like receptors (TLRs) by invading pathogen or pathogenic molecules, such as LPS, Pam2CSK4, or Pam3CSK4, plays a vital role in sepsis pathogenesis [6,7]. Despite dramatic advances in sepsis pathophysiology and treatment procedures, resuscitative strategies for gram-negative sepsis have not been approved to date. Therefore, the search for a novel class of antibiotic agents that can combat and overcome resistant events related to sepsis is urgently required.

Numerous bioactive natural peptides from various organisms play a beneficial role either directly or indirectly in human physiology including antimicrobial and immunomodulatory activities [8,9]. In all species of life, antimicrobial peptides (AMPs) form a component of the innate immune defense. AMPs show broad spectrum potential as small molecule antibiotics [10,11,12]. Persistent infection has resulted in living organisms evolving many host defensive mechanisms against pathogens, including transcription of AMP encoding genes [13]. With over four decades of peptide research, AMPs have emerged as an alternative class of naturally occurring antibiotics. Because AMPs have diverse structures and modes of action with broad spectrum antibacterial activities, development of novel AMPs is a promising strategy for combating multidrug-resistant (MDR) bacteria [14,15].

Till date, more than 3000 AMPs have been identified but only seven have been approved by the U.S. Food and Drug Administration (FDA); these include colistin, gramicidin D, daptomycin, vancomycin, oritavancin, dalbavancin, and telavancin, which are either in the market or under clinical development [16,17]. They have different structures and sequence motifs but share a broad spectrum of antibiotic actions. Although the detailed mechanisms underlying the antibacterial activity of AMPs have not been completely understood, they appear to involve transmembrane pore formation or intracellular killing [18]. Colistin is a well-studied antimicrobial cyclic peptide that targets gram-negative bacteria by binding to the negatively-charged LPS layer in the outer membrane and by causing cell death [19]. Besides antimicrobial properties, colistin has been shown to exhibit LPS-degradative and immunomodulatory effects. However, applications of colistin are limited because of its adverse effects [20]. Cathelicidins are another type of AMP that proteolytically activates in response to bacterial infection; they affect the permeability of the bacterial membrane and exhibit bactericidal effects [21]. Cathelicidin-derived LL-37 peptides can promote LPS neutralization and phagocytosis and exhibit immunomodulatory effects by skewing macrophage differentiation toward pro-inflammatory phenotypes [22,23,24]. Furthermore, proteolytic cleavage of LL-37 fragments could trigger additional inflammatory disorders [25].

Thus, controlling selectivity, improving stability, and reducing cytotoxicity-mediated adverse effects are the prime strategies to design potent AMPs. This can be done by optimizing the chemical properties of AMPs, such as cationicity, hydrophobicity, and α-helicity, to enable them to bind to the anionic bacterial membrane and to damage it [26]. Short peptide analogs designed based on natural AMPs with high antimicrobial activity can reduce production cost and can cause low immunogenicity, resulting in the development of potent novel candidates for therapeutic applications. To reduce the cytotoxicity of parent peptides, new peptide antibiotics can be designed by substituting Lys or Arg for hydrophobic amino acids or D-amino acids in the middle of the peptide sequences [27,28].

Defensins constitute a large segment of cationic arginine-rich peptides that exhibit antibacterial activity against both gram-positive and gram-negative bacteria. Protaetiamycine is a 43-amino-acid insect defensin from the beetle *Protaetia brevitarsis* [29]. To design short AMPs, we previously designed 9-mer peptide analogs of protaetiamycine, namely 9Pbw2 (RLWLAIKRR-NH_2_), 9Pbw3 (RLWLAIWRR-NH_2_), and 9Pbw4 (RLWLAWKRR-NH_2_), based on the sequence of RLWLAIGRG-NH_2_ in protaetiamycine by optimizing the balance between hydrophobicity and cationicity of the peptides [30]. The results revealed that the third 9-meric peptide designed from protaetiamycine 9Pbw3, which was named Pro9-3 in this study, and its enantiomer containing two Trp amino acids (Pro9-3D) exhibited potent antibacterial activities along with toxic effects on mammalian cells [31]. However, their antibacterial activities, regardless of standard bacteria including MDR strains, make Pro9-3 and its enantiomer Pro9-3D attractive candidate antibiotic peptides. Therefore, in this study, we aimed to design two novel 10-meric peptides (Pro10-1 and its enantiomer, Pro10-1D) by adding an Arg residue at the N-terminal of Pro9-3 to increase the potency of the peptides against gram-negative pathogens and bacterial cell selectivity. We evaluated their antibacterial and anti-inflammatory activities using in vitro and in vivo models of gram-negative sepsis and showed that Pro10-1D derived from Pro9-3 could act as a promising peptide antibiotic for sepsis caused by MDR gram-negative bacteria.

## 2. Results

### 2.1. Design of Peptides

**Figure 1:**
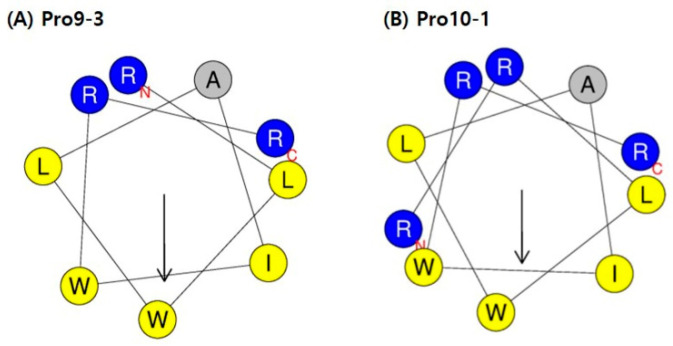
Helical-wheel diagrams of (**A**) Pro9-3 and (**B**) Pro10-1 using HeliQuest online tool [32]: Positively charged amino acid residues are shown in blue, negatively charged residues are in red, and hydrophobic residues are in yellow at the bottom of the wheel. Alanine (A) is shown in gray. The arrows indicate the helical hydrophobic moment.

We had previously designed four 9-meric peptides (Ala22 to Gly30) of the 43-mer insect defensin protaetiamycine [30]. Of the four peptides, only the third analog, Pro9-3 (RLWLAIWRR-NH_2_; previous name, 9Pwb3), and its enantiomeric peptide Pro9-3D showed potent antibacterial activities as well as anti-inflammatory activities, although they also showed high cytotoxicity against mammalian cells [31]. To increase the potency of Pro9-3 and Pro9-3D against gram-negative bacteria as well as to improve their bacterial cell selectivity, we increased the cationicity of Pro9-3 by adding one more Arg at the N-terminus and named this 10-meric peptide Pro10-1. To increase the stability of the peptide, we also synthesized the enantiomeric peptide of Pro10-1 and named it Pro10-1D (Table 1). A helical wheel diagram of Pro9-3 in Figure 1A shows its amphipathicity, although the hydrophobic face at the bottom is much larger compared to the hydrophilic face at the top. Five sequential hydrophobic residues, “LWLAIW”, provide high hydrophobicity (0.776) compared to Pro9-3. In contrast, hydrophobicity of the two newly designed 10-meric peptides was decreased to 0.597 from 0.776 by adding one more Arg. The addition of Arg at the N-terminus reduced the hydrophobic face in the helical wheel of Pro10-1, resulting in a decrease in the hydrophobic moment, a measure of the amphipathicity of an α-helix, from 0.692 to 0.584 (Figure 1B).

### 2.2. Antibacterial Activity

The antimicrobial activities of Pro9-3, Pro9-3D, Pro10-1, and Pro10-1D were examined against a set of representative gram-negative and MDR gram-negative bacterial strains using the micro broth dilution method. Minimum inhibitory concentrations (MIC_100_) was defined as the concentration which kills more than 99% of bacteria. All peptides showed comparable bactericidal properties to those of melittin, which is known to be a potent peptide antibiotic. Pro10-1 showed slightly higher antibacterial activity compared to Pro9-3, implying that an increase of cationicity may promote the interaction between the peptide and the bacterial membrane. Remarkably, Pro10-1D showed much stronger antibacterial activities against all standard gram-negative bacteria compared with Pro10-1 and 9-meric peptides (Table 2). Notably, Pro10-1D treatment showed excellent killing against all gram-negative MDR bacteria when compared to the other Pro-peptides and melittin.

### 2.3. Cytotoxicity of Peptides In Vitro and In Vivo

**Figure 2:**
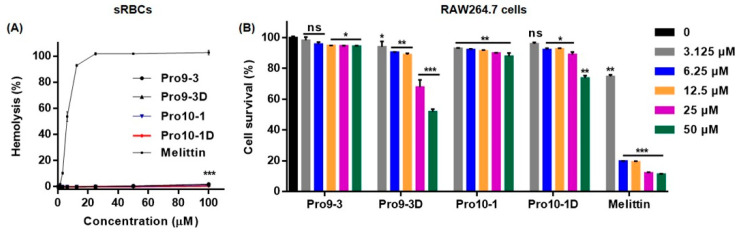
Cytotoxic effects of Pro9-3 and its analogs: (**A**) dose–response curves for sheep red blood cell (sRBC) hemolysis induced by peptides and (**B**) dose-dependent (0–50 μM) cytotoxicity of the peptides against mouse macrophage RAW264.7 cells for 24 h. Melittin was used as a control. The values are expressed as the mean ± SEM of three independent experiments and are statistically significant at * *p* < 0.05, ** *p* < 0.01, and *** *p* < 0.001; ns, not significant (by *one-way ANOVA*, *Tukey’s post hoc test)*.

To determine whether the peptides induce a cytotoxic effect, we first analyzed the hemolytic activity of Pro9-3 and its analogs-Pro9-3D, Pro10-1, and Pro10-1D against sheep red blood cells (sRBCs) (Figure 2A). Incubation of sRBCs with 100 μM Pro9-3 and Pro9-3D peptides induced 2% hemolysis; however, no hemolytic activity was observed with Pro10-1D and Pro10-1 peptides. In contrast, melittin exhibited 100% hemolysis at 25 μM. As listed in Table 2, Pro10-1D showed a higher relative selective index followed by Pro9-3D, Pro10-1, and Pro9-3 (41.18, 31.82, 13.46, and 10.94, respectively) against gram-negative bacteria. Therefore, Pro10-1D exhibited greater bacterial selectivity than the other peptides.

We next investigated the cytotoxicity of Pro-peptides against the murine macrophage cell line RAW264.7. Pro9-3 resulted in 95% and 94% survival rates of RAW264.7 cells when compared with the melittin control (12% and 11.2%) at concentrations of 25 and 50 μM, respectively, whereas Pro9-3D showed a lethal effect with 68% and 51.8% survival rates, respectively (Figure 2B). In contrast, RAW264.7 cells treated with Pro10-1D, which showed higher antibacterial activity than the other peptides, exhibited 89% and 73% survival rates in response to 25 and 50 μM treatments, respectively, when compared with Pro10-1 (90% and 87%). Notably, except for Pro9-3D, all other peptides exhibited an 85% survival rate at 25 μM. Even though Pro9-3D, which has high hydrophobicity, showed high antibacterial activity against MDR gram-negative bacteria, it is very toxic to mammalian cells; thus, it cannot be an effective candidate peptide antibiotic. Therefore, we performed further experiments on the parent peptide, Pro9-3, as well as on newly designed peptides Pro10-1 and Pro10-1D.

### 2.4. Circular Dichroism (CD) Measurement of Peptide Structure in a Membrane Environment

**Figure 3:**
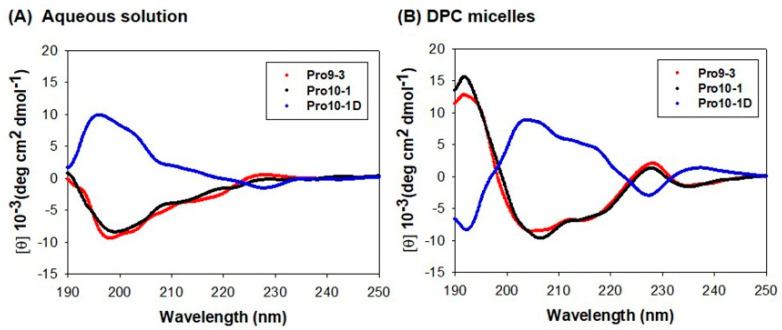
Circular dichroism spectra of Pro9-3 and its analogs at 100 μM in (**A**) aqueous solution and (**B**) 50 mM dodecylphosphocholine (DPC) micelles acquired with 10 scans: Double negative maxima at 205 and 220 nm are characteristic of α-helical structures.

We investigated the secondary structures of peptides in aqueous solution and in membrane-like environments by analyzing their CD spectra. All peptides showed unordered structures in aqueous solution but exhibited conformational changes in dodecylphosphocholine (DPC) micelles (Figure 3). Because Pro-peptides have two Trp residues, the Trp chromophoric side chain can contribute to the CD spectra in the 220–230 nm region. However, in the 220 nm region, the CD spectra of all peptides in DPC micelle clearly showed double negative maxima or minima at 205 nm and 220 nm, suggesting that they may be significantly folded in an α-helical structure in DPC micelles. As Pro10-1D is an enantiomer of Pro10-1, its CD spectra is the mirror image of its parent peptide.

### 2.5. Structural Changes Observed by Chemical Shift Perturbation in Membrane Environments

**Figure 4:**
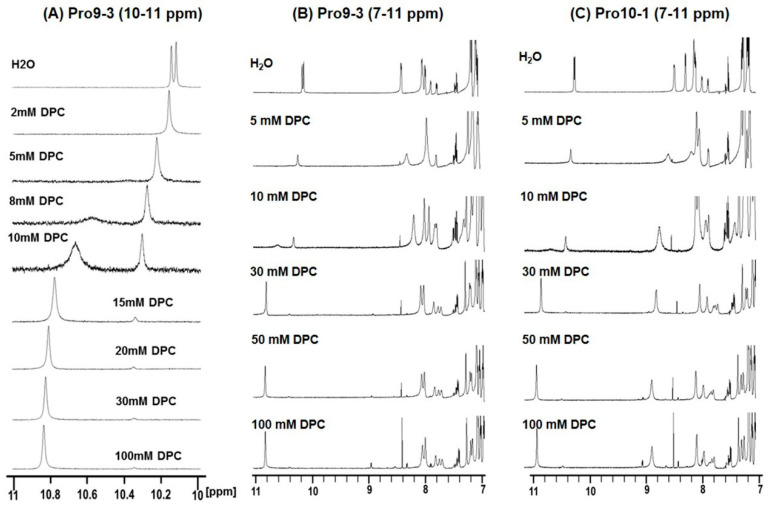
Chemical shift perturbations in the NH region of the ^1^H NMR spectra at 298 K in 9:1 (*v*/*v*) H_2_O/D_2_O induced by dodecylphosphocholine (DPC): Sets of 1D ^1^H NMR spectra of a 1 mM solution of peptides were acquired with 128 scans. One-dimensional ^1^H NMR spectra of Pro9-3 were recorded with 9 different concentrations of DPC from 10 to 11 ppm (**A**), and 1D ^1^H NMR spectra of Pro9-3 (**B**) and Pro10-1 (**C**) from 7 to 11 ppm were recorded for six different concentrations of DPC. Changes in chemical shifts and line broadenings in the spectra of the peptides upon addition of DPC indicate conformational changes and interactions with DPC.

Changes in chemical shifts and line broadenings in the 1D ^1^H nuclear magnetic resonance (NMR) spectra of Pro9-3 and Pro10-1 at 298 K in a 9:1 (*v*/*v*) H_2_O/D_2_O solution (pH 4.5) upon the addition of DPC were investigated (Figure 4). In aqueous solution, all peptides in the free form showed two resolved peaks for the indole rings of the two Trps at 10.2 ppm. As shown in the NMR spectra of Pro9-3 (Figure 4A), the addition of different concentrations of DPC resulted in a downfield shift of indole protons of Trp from the 10 ppm to 11 ppm regions. The addition of DPC to the peptides in water caused conformational changes of peptides because of the interaction with the DPC, resulting in line broadening as well as downfield shifts. At 10 mM DPC, NMR spectra clearly showed two resolved broad peaks for two forms, free form and bound form, in slow exchange. However, at 15 mM DPC, the indole proton peak at downfield for the bound form became sharper compared to those with lower amounts of DPC while the peak for the free form exhibited a decrease in intensity. After the addition of 30 mM DPC, chemical shift as well as line broadening did not change, implying that peptides are bound stably to the DPC micelles (Figure 4B,C). Even though Pro10-1 has one more Arg compared to Pro9-3, the overall patterns of chemical shift changes were very similar in both cases. Pro10-1D, an enantiomer of Pro10-1 with all D-amino acids, showed almost the same NMR spectral changes as Pro10-1 upon addition of DPC (data not shown). Furthermore, large chemical shift perturbations in the amide protons and the aromatic ring regions were also observed for all peptides (Figure 4B,C). These changes imply that Pro-peptides have random structures in aqueous solution, whereas they form more ordered structures in micellar environments, which agrees well with the CD spectra (Figure 3)

### 2.6. Mechanism of Antibacterial Activities Against Gram-Nnegative Bacteria

**Figure 5:**
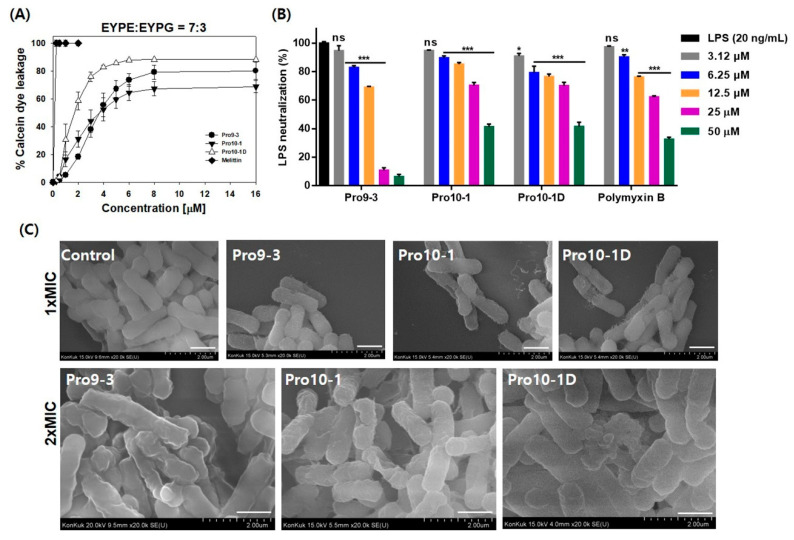
Antibacterial mechanism of peptides: (**A**) Dose–response curves of calcein leakage from egg yolk L-α-phosphatidylethanolamine (EYPE)/egg yolk L-α-phosphatidylglycerol (EYPG) (7:3, w/w) large unilamellar vesicles (LUVs) (induced by the peptides) were constructed. The concentration-dependent dye release indicates the membrane-permeabilizing ability of the peptides. Melittin was used as a control. (**B**) Lipopolysaccharide (LPS)-neutralizing capacity of the peptides and polymyxin B (positive control): the percentage of inhibition signifies the LPS-neutralizing abilities of peptides. (**C**) Scanning electron micrographs of *E. coli* (KCTC 1682) treated with peptides at 1× and 2 × of the minimum inhibitory concentrations (MICs) for 4 h: ultrastructural changes to the outer membrane of *E. coli* indicate the membrane damage caused by the peptides. Scale bar, 100 μm. Data are expressed as the mean ± SEM of three independent experiments and are statistically significant at * *p* < 0.05, ** *p* < 0.01, and *** *p* < 0.001; ns, not significant (by *one-way ANOVA*, *Tukey’s post hoc test*).

We investigated the modes of action of the peptides by measuring their abilities to permeate model phospholipid membranes and *Escherichia coli*. The membrane-permeabilizing ability of the peptides was measured by monitoring the release of fluorescent calcein from liposomes of various compositions. We employed negatively-charged large unilamellar vesicles (LUVs) composed of 7:3 (*w*/*w*) egg yolk L-α-phosphatidylethanolamine (EYPE)/egg yolk L-α-phosphatidylglycerol (EYPG) to mimic gram-negative bacterial cells. The percentage of calcein leakage 3 min after peptide exposure was used to assess its ability to permeate the membrane. Figure 5A shows the dose–response relationship of the peptide-induced calcein release. All Pro-peptides induced significant dye release against the negatively charged EYPE/EYPG LUVs, implying that they potently permeate bacterial cell component membranes. Especially, Pro10-1 and Pro10-1D peptides showed more than 80% leakage, even at 4 µM, whereas Pro9-3 showed 64.7% leakage at 4 µM. These results suggest that the potent antibacterial activities of these peptides may be due to permeabilization of the bacterial cell membrane. Therefore, the newly designed peptides, Pro10-1 and Pro10-1D with higher cationicity, which retained high antibacterial activity similar to that of melittin, effectively interact with the membrane to cause fatal damage to the bacterial cells, resulting in improved antibacterial activities.

### 2.7. LPS-Neutralizing Activities

The capacity of Pro-peptides to neutralize LPS was evaluated using limulus amebocyte lysate (LAL) assays. As shown in Figure 5B, all Pro-peptides significantly interacted with LPS in a concentration-dependent manner. At 50 μM, Pro9-3, Pro10-1, and Pro10-1D peptides notably inhibited the activation of LPS by 93.6%, 58.6%, and 58.45%, respectively, when compared to the positive control polymyxin B (67.4%), with Pro9-3 showing much greater affinity for LPS.

### 2.8. Visualization of the Effects of Pro-Peptides on Morphology of E. coli by Scanning Electron Microscopy

We next investigated the interaction between Pro-peptides and bacteria by field emission scanning electron microscopy (FE-SEM). Membrane damage induced by peptide treatment was clearly visualized, as shown in Figure 5C. *E. coli* cells treated with a 1× MIC of the peptides for 4 h were wrinkled, whereas the control cells without peptide treatment had a normal oval shape. *E. coli* cells treated with 2 × MIC of all peptides were even more crumpled and severely contracted. These results proved that the Pro-peptides effectively permeabilized the membrane to cause severe damage to *E. coli*.

### 2.9. Resistance of Peptides Against Protease Digestion

**Figure 6:**
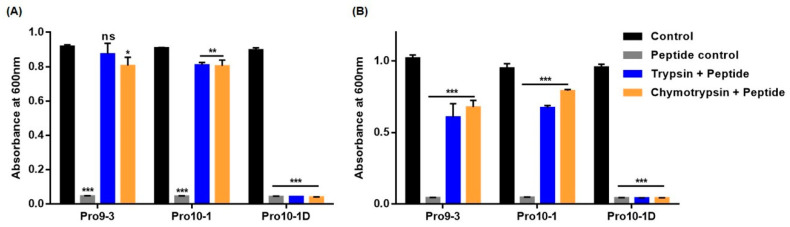
Inhibition of antimicrobial activity of peptides by trypsin and chymotrypsin assessed using the broth microdilution method: peptides at their respective minimum inhibitory concentrations were preincubated with trypsin and chymotrypsin for 6 h, and the treated peptides were examined for their antimicrobial activity against (**A**) *E. coli* and (**B**) *A. baumannii* for 16 h. Inhibited bacterial growth signifies the proteolytic stability of peptides. Data are shown as the mean ± SEM of three independent experiments and are statistically significant at * *p* < 0.05, ** *p* < 0.01, and *** *p* < 0.001; ns, not significant (by *one-way ANOVA*, *Tukey’s post hoc test*).

Rapid cleavage of AMPs by endogenous proteases is a major limitation for the clinical use of AMPs [28]. To test the stability of Pro10-1 and Pro10-1D, we challenged the peptides with proteases trypsin and chymotrypsin, respectively, and then, the relative survival rates of *E. coli* and *A. baumannii* were determined (Figure 6). As shown in Figure 6A,B, the growth of the aforementioned bacteria was not inhibited by Pro9-3 and Pro10-1 at concentrations of up to 64 μM in the presence of both proteases whereas enantiomeric Pro10-1 retained its bactericidal activity and significantly inhibited the growth of *E. coli* and *A. baumannii*, similar to the case without the proteases. This result revealed that Pro10-1D showed substantial protease resistance against trypsin and chymotrypsin.

### 2.10. Effect of Peptides on Biofilm Inhibition

**Figure 7:**
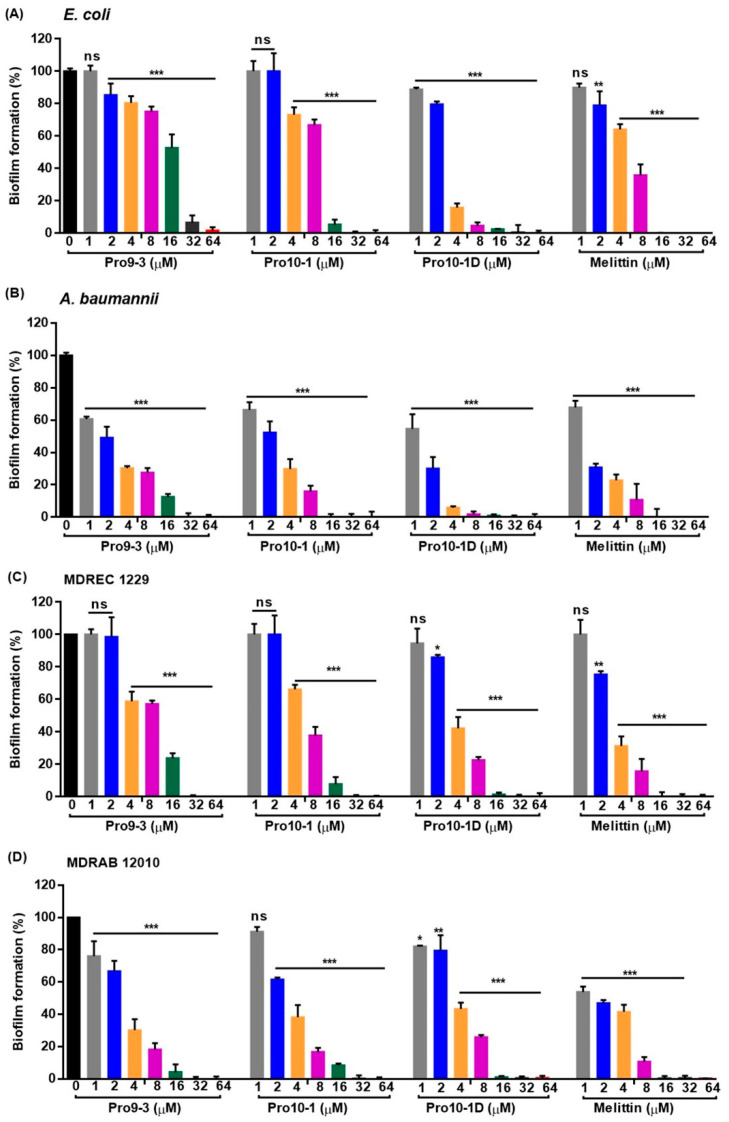
Inhibitory effects of Pro9-3, Pro10-1, and Pro10-1D on bacterial biofilm formation: Biofilm degradation properties of peptides were measured against (**A**) *E. coli*, (**B**) *A. baumannii*, (**C**) MDREC 1229, and (**D**) MDRAB 12010 in a dose-dependent manner. Melittin was used as a control. Data are shown as the mean ± SEM (*n* = 3) and are statistically significant at * *p* < 0.05, ** *p* < 0.01, and *** *p* < 0.001; ns, not significant by *one-way ANOVA*, *Tukey’s post hoc test*.

We next investigated the antibiofilm potential of Pro9-3, Pro10-1, and Pro10-1D using crystal violet staining. As shown in Figure 7, all Pro-peptides inhibited biofilm formation in a concentration-dependent manner. Particularly, Pro10-1D was the most potent peptide for inhibiting biofilm formation by *E. coli* and *A. baumannii* as well as their MDR strains compared to the other peptides, including melittin. Pro10-1D inhibits the growth of MDREC 1229 completely at 4 μM in Mueller–Hinton (MH) media, as shown in Table 2. However, when we added glucose (0.2%) to induce biofilm formation of MDREC 1229 on the surface of polystyrene plates, Pro10-1D inhibited the biofilm formation by 58% at 4 μM and by 99% at 16 μM.

**Figure 8:**
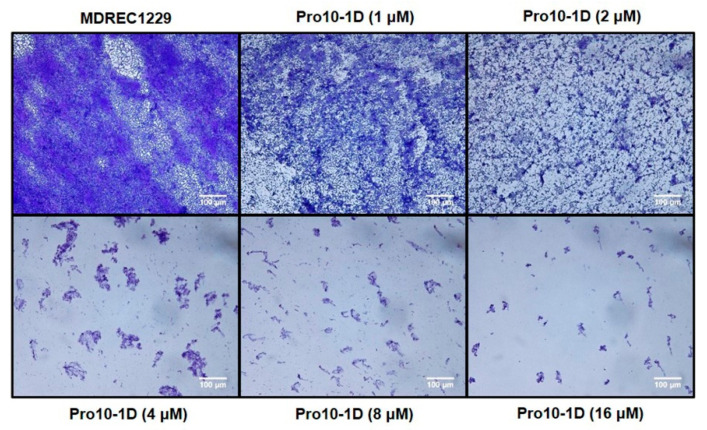
Light microscopic images representing the antibiofilm effect of Pro10-1D against MDREC 1229: MDREC 1229 cells were allowed to adhere the polystyrene surface of the culture plate and were then exposed to Pro10-1D (0–16 μM) for 16 h. Strains were cultured in Mueller–Hinton (MH) media, and biofilm formation was evaluated by crystal violet staining. Pro10-1D-exposed biofilms appeared thinner and scantier than untreated MDREC 1229; these effects were dose-dependent. Scale bar, 100 μm.

Since Pro10-1D showed the highest potency for inhibiting biofilm formation among three peptides, the antibiofilm potential of Pro10-1D against MDREC 1229 was further confirmed through light microscopy (Figure 8). The addition of glucose in Mueller-Hinton (MH) media caused biofilm formation of MDREC 1229, and a dense layer of polymeric matrix was observed in MDREC 1229. In contrast, Pro10-1D-exposed cells showed a disrupted biofilm matrix with less bacterial colonies; these effects depended on the concentrations of Pro10-1D. These results confirmed the antibiofilm potential of Pro10-1D. Because Pro10-1 and Pro10-1D showed higher antibacterial as well as anti-biofilm activities than Pro9-3, we next examined the anti-inflammatory activities of these two newly designed peptides to gain more insights into the mechanism of their anti-inflammatory response.

### 2.11. Suppression of Inflammatory Cytokine Expression Levels in LPS-Stimulated RAW264.7 Cells

**Figure 9:**
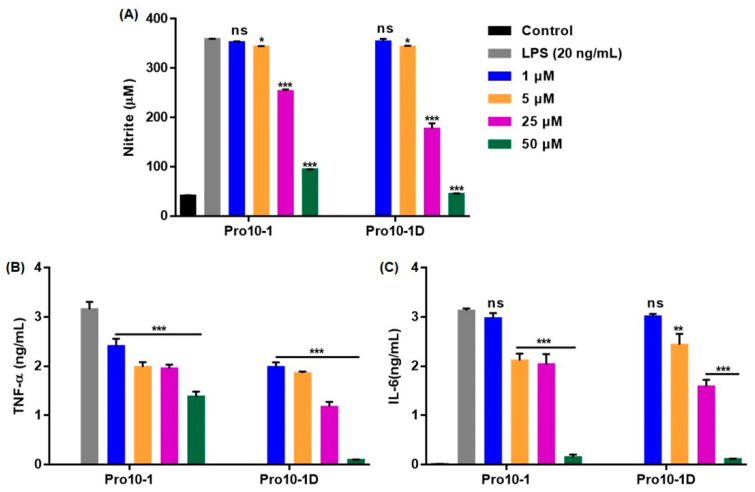
Pro10-1D modulates lipopolysaccharide (LPS)-induced inflammatory response in RAW264.7 cells. (**A**) Graphical data signify the dose-dependent inhibition of nitrite production and (**B**,**C**) expression of inflammatory cytokines (TNF-α and IL-6) in RAW264.7 cells incubated with peptides (0–50 μM) and then challenged with LPS (20 ng/mL) for 16 h. Data are expressed as the mean ± SEM of three independent experiments and are statistically significant, based on *one-way ANOVA* (*Tukey’s post hoc test*, * *p* < 0.05, ** *p* < 0.01, and *** *p* < 0.001; ns, not significant).

The LPS released from pathogens stimulate host immune cells such as macrophages and fibroblast to produce cytokines that can ultimately lead to septic-related diseases [33]. Thus, we examined the inhibitory effect of the peptides on nitric oxide (NO) secretion, followed by ELISA-based measurement of cytokine levels (including Tumor necrosis factor alpha (TNF-α) and Interleukin 6 IL-6) in LPS-stimulated RAW264.7 macrophage cells. As expected, LPS-challenged RAW264.7 cells showed substantially elevated NO, TNF-α, and IL-6 levels (Figure 9). However, treatment with Pro10-1 and Pro10-1D showed effective inhibition of these LPS-induced changes, even at 25 μM when compared with the 50 μM treatment. Furthermore, Pro10-1D significantly inhibited NO levels along with a downregulation of TNF-α and IL-6 expression in LPS-treated cells when compared with Pro10-1. These results specified that Pro10-1D can downregulate LPS-induced cytokine levels efficiently.

### 2.12. Pro10-1D Targets TLR4-Induced NF-κB and MAPK Signaling in RAW264.7 Cells

**Figure 10:**
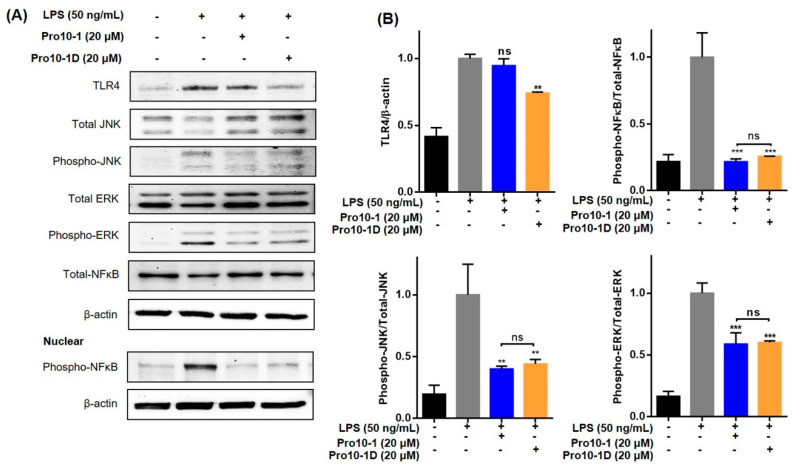
Pro10-1D is a target of toll-like receptor 4 (TLR4) and downregulates MAPK and NF-κB translocation in LPS-stimulated RAW264.7 cells. (**A**) Immunoblot analysis revealed the suppression of LPS-mediated TLR4, total/phospho-JNK, total/phospho-ERK, and cytosolic and nuclear translocation of NF-κB in RAW264.7 cells treated with peptides (20 μM) and LPS (50 ng/mL). β-actin was used as an internal control. (**B**) Graphical data represent the densitometry analysis of respective proteins, quantified by ImageJ. The values are expressed as the mean ± SEM of three independent experiments and are statistically significant, based on *one-way ANOVA* (*Tukey’s post hoc test*, ** *p* < 0.01, and *** *p* < 0.001; ns, not significant).

LPS of gram-negative infection is recognized by the TLR4 receptor and subsequently leads to the production of several downstream transcription factors, including NF-κB, which contributes to pathogenesis of several inflammatory diseases [34]. To understand the effect of the newly designed peptides on TLR4 regulation, we analyzed the expressions of TLR4 and mitogen-activated protein kinase (MAPK) ( c-Jun N-terminal kinase (JNK) and Extracellular signal-regulated kinase (ERK)) and NK-κB nuclear translocation in LPS-stimulated RAW264.7 cells via immunoblot assays (Figure 10). LPS treatment increased the expression of TLR4, followed by significant phosphorylation of MAPK and NF-κB translocation in RAW264.7 cells. Treatment with 20 μM Pro10-1 and Pro10-D effectively suppressed LPS-induced TLR4 expression as well as downregulated MAPK and NF-κB proteins. A comparative analysis showed that Pro10-1D showed improved downregulatory effects compared to Pro10-1. Hence, we suggest that Pro10-D could influence the inflammatory cues by inhibiting TLR4-mediated cascades.

### 2.13. In Vivo Cytotoxicity Profile of Pro10-1D

**Figure 11:**
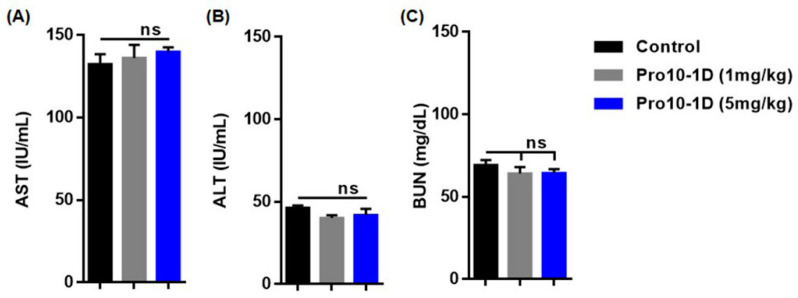
Pro10-1D exerts nontoxic effects in vivo. The representative graph indicates (**A**) aspartate aminotransferase (AST), (**B**) alanine aminotransferase (ALT), and (**C**) blood urea nitrogen (BUN) levels of mice treated with Pro10-1D (1 mg/kg and 5 mg/Kg) for 24 h. Control mice only received phosphate-buffered saline. Data are presented as the mean ± SEM (5 mice/group). Statistical analysis was performed by *one-way ANOVA* (*Tukey’s post hoc test*; ns, not significant).

As Pro10-D showed the highest antibacterial and antibiofilm activities along with anti-inflammatory activities, we selected Pro10-1D as a potential candidate for conducting experiments with the in vivo sepsis model. To examine its potency for therapeutic application to gram-negative sepsis, we examined the safety of Pro10-1D in mice. We first intraperitoneally injected mice with a single dose of Pro10-1D (1 mg/kg and 5 mg/kg) and evaluated its toxicity in vivo for 24 h. We analyzed the serum levels of marker enzymes in the liver (aspartate aminotransferase (AST) and alanine aminotransferase (ALT)) and kidney (blood urea nitrogen (BUN)) in mice. We did not observe any clinical significance of AST, ALT, and BUN elevation at both doses (Figure 11), confirming that the peptide was safe even at high doses. Therefore, we selected the 1 mg/kg dose of Pro10-1D in the murine model of sepsis.

### 2.14. Antisepsis Effect of Pro10-1D Peptide on E. coli K1-induced Mouse Model of Septic Shock

**Figure 12:**
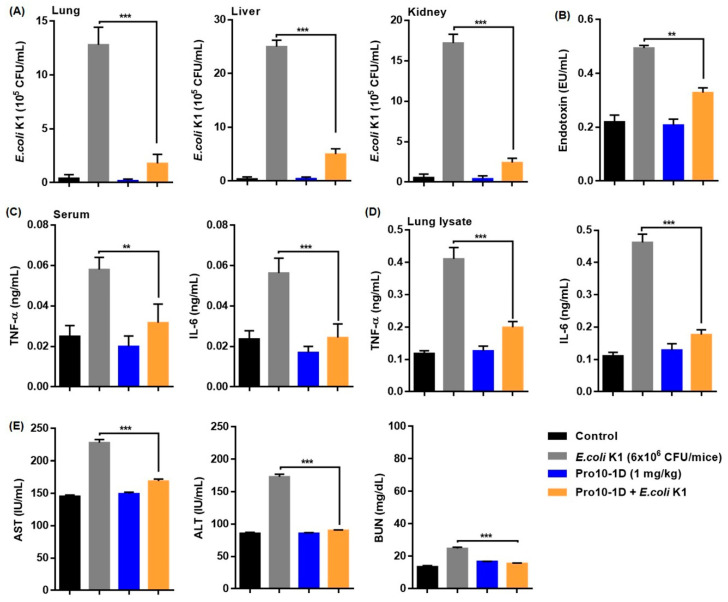
Pro10-1D treatment alleviates septic shock in *E. coli* K1-infected mice: (**A**) Inhibition of bacterial growth by Pro10-1D in the lung, liver, and kidney lysates of the septic mice. Mice were pretreated with 1 mg/kg of Pro10-1D (1 h) and *E. coli* K1 (6 × 10^6^ Colony-Forming Unit (CFU)/mice, 16 h). (**B**) Pro10-1D reduces circulating endotoxin (LPS) levels in serum of septic mice. Effect of Pro10-1D on TNF-α and IL-6 levels in the (**C**) serum, and (**D**) lung lysates of septic mice. (**E**) Pro10-1D normalizes the serum levels of (**E**) aspartate aminotransferase (AST), alanine aminotransferase (ALT), and blood urea nitrogen (BUN) in septic mice infected with *E. coli* K1. The graphs indicate the mean ± SEM (5 mice/group). ** *p* < 0.01; *** *p* < 0.001; ns, nonsignificant (by *one-way ANOVA*, *Tukey’s post hoc test*).

We next examined the potency of Pro10-1D as a candidate antiseptic peptide drug using an effective septic shock mouse model established using *E. coli* K1. Pro10-1D reduced the number of bacteria in the mouse lung, liver, and kidney by approximately 73%, 87%, and 71%, respectively. This indicated that Pro10-1D can effectively inhibit bacterial growth in visceral organs of *E. coli* K1-infected mice (Figure 12A). Furthermore, serum endotoxin measurement by the LAL test (Figure 12B) revealed that *E. coli* K1-infected mice exhibited 100% endotoxin activation, whereas pretreatment with Pro10-1D effectively reduced the endotoxin levels by over 33%. We also quantified the inflammatory cytokine levels (TNF-α and IL-6) using ELISA to determine the suppressive effect of Pro10-1D. As shown in Figure 12C,D, *E. coli* K1-challenged mice exhibited significantly upregulated TNF-α and IL-6 levels compared with the control. Treatment with Pro10-1D evidently suppressed TNF-α and IL-6 levels by 45% and 56%, respectively, in serum and by 51% and 62%, respectively, in lung tissue of septic mice. Considering the role of sepsis in multiple-organ failure, we analyzed the serum levels of marker enzymes in the liver and kidney. As expected, *E. coli* K1-infected mice displayed 100% enhanced AST, ALT, and BUN levels compared to the control. However, the mice pretreated with Pro10-1D showed significant reduction of the enzyme levels to 26% for AST, 48% for ALT, and 37% for BUN (Figure 12E).

### 2.15. Pro10-1D Treatment Suppresses Polymorphonuclear Lymphocyte (PMN) Infiltration in E. coli K1-Induced Mouse Model

**Figure 13:**
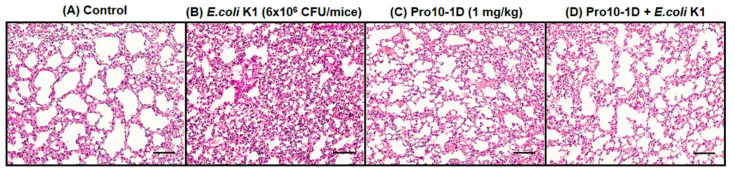
Pro10-1D treatment ameliorates lung microanatomical changes in *E. coli* K1-infected mice. Representative hematoxylin and eosin staining of lung tissue of (**A**) the control shows normal lung anatomy, while that of (**B**) *E. coli* K1-infected mice (6 × 10^6^ CFU/mice, Intra-peritoneal injection (I.P.)) displays severe lung damage associated with neutrophil infiltration. (**C**) No sign of lung damage can be observed in the Pro10-1D-treated control mice (1 mg/Kg, I.P.), while (**D**) Pro10-1D pretreatment distinctly ameliorated the aberrant changes induced by *E. coli* K1 infection (×20 magnification, scale bar, 100 μm).

Histological patterns of *E. coli* K1-infected lung tissues displayed severe edema, pulmonary congestion, and alveolar hemorrhage, along with infiltrated PMNs in the lungs (Figure 13). These microanatomic features validate that increased PMNs are associated with lung damage, leading to sepsis severity upon *E. coli* K1 infection. Pro10-1D effectively alleviated the pathological changes induced by *E. coli* K1 in septic mice. Hence, we suggest that Pro10-1D can restore *E. coli*.

K1 induced lung damage by suppressing the entry of PMNs at the site of infection. Overall, we speculate that the Pro10-1D peptide can act as a novel peptide antibiotic in the prevention of sepsis-related inflammatory insults in *E. coli* K1-infected mice.

## 3. Discussion

AMPs have superior benefits over conventional antibiotics in terms of MDR resistance and the broad spectrum of antibacterial activities. Furthermore, their ability to modulate the host immune response and to neutralize LPS proved the potency of AMPs as powerful agents for the treatment of bacterial infections [35]. Particularly, gram-negative infections are serious public concerns due to MDR bacteria, and the development of novel peptide antibiotics against gram-negative bacteria is of critical priority [36]. Many AMPs designed from potent natural peptides from various organisms have shown therapeutic potentials for clinical application. Some AMPs are in the development stages of clinical trials. For example, hLF1-11 derived from human lactoferrin is in phase 1 trials [37], and P-113 derived from human histatin-5 [35,38] and Omiganan derived from indolicidin are in phase 3 trials [39]. Among these, hLF1-11 and P-113 show potency against gram-negative infections [40,41]. However, these AMPs also have limitations for commercial and clinical applications such as weak activities, nonspecific cytotoxicity, and poor pharmacokinetic properties. For example, pexiganan (MSI-78) derived from magainin developed as a topical agent failed because it was not superior to other treatment methods [42].

To improve the therapeutic potential of AMPs against gram-negative bacteria, it is necessary to optimize the properties of AMPs [43]. Increased net charge can enhance antimicrobial activity through electrostatic interactions with the negatively charged gram-negative bacterial membranes. Pseudin is an AMP derived from frog skin which shows high antibacterial activity against gram-negative bacteria with high cytotoxicity [44]. Optimal balance between cationicity and hydrophobicity is important for antibacterial activity and cytotoxicity against mammalian cells [45]. Lys substitutions improved the cell selectivity of pseudin, but excessive positive charge by Lys substitution also increased cytotoxicity [46]. Substitution of Lys for Leu18 in pseudin showed antiseptic activities against gram-negative sepsis [47].

Polymyxin B and colistin are cyclic cationic lipopeptide antibiotics, which specifically target the bacterial membrane of gram-negative bacteria. Even though their clinical use is limited by nephrotoxicity due to the threat of MDR gram-negative bacteria, they serve as the “last resort” therapeutic option against MDR gram-negative bacteria [48]. Therefore, researchers have attempted to develop derivatives of polymyxin to improve its properties. Analogs of polymyxin, SPR741, SPR1205, SPR206, and SPR946 have been designed by shortening the peptide chains, by modifying the acyl chains, and by substituting the amino acid residues to reduce nephrotoxicity and to improve potency [49,50,51,52,53,54,55]. SPR206 is a novel polymyxin B analog for the treatment of gram-negative infection that is undergoing a phase I clinical trial [56].

Substitutions of L-amino acid with D-amino acid and the introduction of peptoid or proline (Pro) in the middle of an α-helical structure can induce bent structures, resulting in reduction of cytotoxicity of AMPs [56,57,58,59]. Enantiomerization, PEGylation, lipidation, and cyclization can enhance the bioavailability of AMPs and their specificity toward gram-negative bacterial membranes [60,61]. These variable methods are effective ways to increase the selectivity of AMP against the gram-negative bacterial membrane, resulting in increased therapeutic potential. There are many potent natural AMPs with long amino acid sequences, but their use may be limited due to their length. For example, papiliocin with 37 amino acids shows high antibacterial activity against gram-negative bacteria; however, 37 residues can make the production costs high [61]. Previously, we designed 12-meric AMP analogs from the N-terminus of papiliocin with antiseptic activity against gram-negative infections [62,63].

In this study, we intended to develop novel short AMPs that show higher cell selectivity than Pro9-3. We showed that the addition of single cationic amino acid (Arg) and the incorporation of D-form into Pro9-3 induced a substantial improvement in its antibacterial and anti-inflammatory activities with cell selectivity. An increase in the cationicity of Pro10-1 may promote electrostatic interactions between the positively charged side chains and the negatively charged phospholipid of the bacterial membrane, resulting in stronger antimicrobial activity against gram-negative bacteria than the parent peptide Pro9-3.

Furthermore, the enantiomeric analog, Pro10-1D, showed higher antibacterial activity and superior relative selective index when compared to Pro10-1, implying that different chirality-related interactions may be important. Cell surface oligosaccharides with different chirality play critical roles in biological recognition. They are also important determinants of the recognition between the membrane and the peptides [64]. Therefore, enantiomeric peptides can have different stereospecific interactions with the oligosaccharides of LPS and the bacterial membrane. Therefore, D-amino acids have been introduced into many AMPs. These may be the important reason for the strong susceptibility of gram-negative bacteria to Pro10-1D. The exact mechanism needs to be verified further.

Several reports have stressed that the incorporation of D-amino acids can improve the proteolytic stability of peptides [65]. Our result showed that enantiomerization of Pro10-1 with D-amino acids improved the proteolytic stability towards trypsin and chymotrypsin in Pro10-1D and improved the antibacterial activity. The proteolytic stability of D-amino acids is also supported by Casciaro et al., who found that Esculentin-1a(1-21)NH2, an analog of esculentin-1 designed by introducing D-amino acids and the covalent conjugation of the peptide to gold nanoparticles, showed powerful antimicrobial activities against gram-negative bacteria and immunomodulatory properties [66].

Our CD spectral data and NMR spectra (Figure 3 and Figure 4) suggest that both Pro10-1 and Pro10-1D adopted an α-helical conformation like that of the parent Pro9-3 in DPC micelle, which mimics the amphipathic environment of a phospholipid bilayer. Additionally, dye-leakage and electron microscopy evidence successfully revealed that the newly designed Pro-peptides may permeabilize the negatively charged bacterial membrane efficiently with the mechanism. It was not possible to determine the solution structure of these peptides due to severe spectral overlaps and the lack of nuclear Overhauser effects. However, our results confirmed the potency of the novel peptide antibiotics and the mechanism of membrane-disrupting properties of the peptides.

Microbial biofilms are polymeric aggregates that result in increased patient mortality and pose adaptive resistance to conventional antibiotics [67]. *E. coli, A. baumannii*, and other MDR strains have the ability to colonize biotic (e.g., skin, mucosa, and wounds) and abiotic materials (e.g., catheters), leading to chronic infections [68]. Another probable mechanism responsible for bacterial biofilm formation is the stringent response characterized by growth arrest and modulation of gene expression under hostile environments [69]. In our study, we found that Pro10-1D inhibited biofilm formation and disrupted the mature biofilms of MDR strains (MDREC 1229 and MDRAB 12010). Recently, Andresen et al. validated that the innate defense regulator peptide-1018 (IDR-1018), a derivative of bactenecin, specifically targets gram-negative and MDR strains by inhibiting the signaling nucleotide (p)ppGpp, which is involved in stringent response and biofilm formation [70]. Another study also stated that Trp/Arg-containing AMPs can kill susceptible cells [71]; thus, direct targeting of cell membrane and/or the modulatory effect of stringent signaling by Pro10-1D might be the possible mechanism responsible for its potent antibiofilm activity.

Aberrant cytokine production is the primary mediator of inflammation-mediated septic shock [72]. Typically, recognition of LPS by the TLR4-MD2 complex results in the recruitment of myeloid differentiation factor 88 (MyD88), resulting in the activation of mitogen-activated protein kinases (MAPKs) and transcription factor NF-κB; this promotes the production of several pro-inflammatory cytokines involved in sepsis [73]. Pro10-1D effectively inhibited LPS and suppressed inflammatory cytokine production via TLR4-mediated NF-κB signaling in LPS-stimulated RAW264.7 cells in vitro. Recent studies have reported that AMPs such as lactoferrin, LL-37, and its analogue KR-12-a5 triggers anti-inflammatory effects by effectively inhibiting the release of LPS-induced inflammatory cytokines by macrophage cells [74,75]. Our results are consistent with those reported in these studies, indicating that Pro-peptides may not only contribute to the antibacterial activities against gram-negative bacteria but also display potent anti-inflammatory activities. Bacterial clearance in sepsis is vital for patient survival. In this study, Pro10-1D-treated mice showed increased bacterial clearance as evidenced by the significant inhibition of bacterial growth in major visceral organs. In addition, Pro10-1D exhibited potent endotoxin-neutralizing activity in vivo, thereby suppressing the production of TNF-α and IL-6 along with significant recovery of lung infections in a septic shock mouse model. The in vivo antiseptic ability could make Pro10-1D an alternative therapeutic candidate for gram-negative sepsis.

The above findings show that Pro10-1D exerts stable proteolytic resistance and potent antimicrobial activity against standard as well as MDR strains. Additionally, Pro10-1D effectively neutralized LPS and suppressed inflammatory cytokine production via TLR4-mediated NF-κB signaling in LPS-stimulated RAW264.7 cells in vitro. Taken together, along with its in vivo antiseptic effects, Pro10-1D could serve as a promising novel antimicrobial candidate for controlling MDR gram-negative bacterial infection that induces septic shock.

## 4. Materials and Methods

### 4.1. Materials

LPS purified from *Escherichia coli* O111:B4, sodium dodecyl sulfate (SDS), radioimmunoprecipitation assay buffer (RIPA), acrylamide, calcein, Hematoxylin and Eosin (H&E), polyvinylidene difluoride (PVDF) membrane, and horseradish peroxidase (HRP)-conjugated anti-rabbit IgG or anti-mouse IgG were purchased from Sigma-Aldrich (St. Louis, MO, USA). Primary antibodies toward TLR4 (ab13556), phospho-NF-κB (#3031), Total NF-κB (#8242), phospho-ERK (#9106), Total-ERK (#9107), phospho-JNK (#4671), Total-JNK (#9252), and β-actin (sc-47778) were purchased from Abcam (Cambridge, MA, USA), Cell Signaling Technology (Danvers, MA, USA) and Santa-Cruz Biotechnology (Dallas, TX, USA). Perdeuterated dodecylphosphocholine (DPC), egg yolk L-a-phosphatidylcholine (PC), and egg yolk L-a-phosphatidyl-DL-glycerol (PG) were purchased from Avanti Polar Lipids, Inc. (Albaster, AL, USA). All other reagents and chemicals used were of high analytical grade.

### 4.2. Peptide Synthesis

All peptides were synthesized by solid-phase synthesis using N-(9-fluorenyl) methoxycarbonyl solid-phase synthesis technique and were purified by reversed-phase preparative high-performance liquid chromatography [76]. Peptide purities were checked by an analytical C_18_ column as >95%, and the molecular masses of the peptides were determined by matrix-assisted laser-desorption ionization-time-of-flight (MALDI-TOF) mass spectrometry at Korea Basic Science Institute (KBSI, Ochang, Korea).

### 4.3. Bacterial Strains

Two strains of gram-negative bacteria, namely *E. coli* (KCTC 1682) and *A. baumannii* (KCCM 40203) from the Korean Collection for Type Cultures (KCTC) (Taejon, Korea) and Korean Culture Center of Microorganisms (KCCM) (Seoul, South Korea). *Escherichia coli* K1 strain RS218 (O18:K1:H7) was a gift from Prof. Jang-Won Yoon in Kangwon National University (Chuncheon, South Korea). Multidrug-resistant gram-negative bacteria (*E. coli* CCARM 1229, *E. coli* CCARM 1238, *A. baumannii* CCARM 12010, and *A. baumannii* CCARM 12220) were obtained from the Culture Collection of Antibiotic-Resistant Microbes (CCARM) at Seoul Women’s University (Seoul, South Korea).

### 4.4. Antimicrobial Assay

The concentration of peptides that completely inhibited (99%) bacterial growth was determined by MIC. Briefly, the microplates were inoculated with two-fold serial dilutions of each peptides in Mueller–Hinton (MH) media and then seeded with log-phase culture of aforementioned bacterial suspensions (2 × 10^5^ CFU/mL). The microplates were incubated for 16 h at 37 °C, and absorbance was read at 600 nm using microplate reader SpectraMAX (Molecular Devices, San Jose, CA, USA).

### 4.5. Hemolytic Activity

The hemolytic activity of all Pro-peptides were tested against sheep red blood cells (sRBCs) as described in previous reports [76]. Fresh sRBCs were washed three times with phosphate-buffered saline (PBS) followed by centrifugation for 5 min at 2500 rpm at 4 °C. Peptides (0.2 to 100 μM) in PBS were incubated with 4% (*v*/*v*) sRBCs for 1 h at 37 °C. The contents were then centrifuged for 5 min at 1000 × g. The absorbance of the supernatant at 405 nm was used as a measure of hemolysis, and three times independent measurements were averaged. As a control, 100% hemolysis was obtained by treating sRBCs with melittin.

### 4.6. Cell Culture and Cytotoxicity Assessment

Murine RAW264.7 macrophage cells were purchased from Korean Cell Line Bank (Seoul, South Korea). The cells were cultured in Dulbecco modified Eagle medium (DMEM) (Thermo Fischer Scientific Inc., MA, USA) supplemented with 10% fetal bovine serum, 100 U/mL penicillin, and 100 mg/mL streptomycin at 37 °C in a humidified 5% CO_2_ incubator. The cytotoxicity of peptides against RAW264.7 cells was determined using WST-8 Cell Proliferation Assay Kit (Biomax Co.,Ltd, Seoul, South Korea). Briefly, cells (1 × 10^5^) were seeded in 96-well plated and then treated with 3.125, 6.25, 12.5, 25, and 50 μM of respective peptides for 24 h followed by addition of 10 μL of the WST-8 reagent for another 3 h incubation at 37 °C. The color intensity was read at 450 nm using a microplate reader.

### 4.7. Circular Dichroism (CD) Analysis

All CD experiments were performed using a J-810 spectropolarimeter (Jasco, Tokyo, Japan) with a 1-mm path length cell at 25 °C. The CD spectra of the peptides at 100 μM were recorded in 0.1-nm intervals from 190 to 250 nm. To investigate the conformational changes induced by membrane environments, CD experiments were performed in aqueous solution and in 50 mM DPC micelles as described previously [62]. Data from 10 scans were averaged for each spectrum and smoothed using J-810. CD data are expressed as the mean residue ellipticity (θ) in deg·cm^2^·dmol^−1^.

### 4.8. Nuclear Magnetic Resonance (NMR) Analysis

Peptides were dissolved in 0.45 mL of a 9:1 (*v*/*v*) H_2_O/D_2_O (pH = 6.0) solution. Perdeuterated DPC was added to the peptide samples. A set of ^1^D-^1^H NMR spectra of peptides was recorded with different concentrations of DPC from 0 to 100 mM. Chemical shifts are expressed relative to the 4,4-dimethyl-4-silapentane-1-sulfonate signal at 0 ppm. All NMR experiments were performed on Bruker 700 Avance spectrometers (Bruker Corporation, Billerica, MA, USA) at the Korea Basic Science Institute (Ochang, South Korea). Data from 128 scans were averaged for each ^1^D-^1^H NMR spectrum. NMR spectra were processed with Bruker Topspin 3.6.0 software (Bruker Corporation, Billerica, MA, USA) and NMRPipe [77].

### 4.9. Calcein Leakage Assay

Calcein-entrapped LUVs composed of egg yolk phosphatidylethanolamine (EYPE)/egg yolk phosphatidylglycerol EYPG (7:3, *w*/*w*) were prepared by vortexing the dried lipid in dye buffer solution (70 mM calcein, 10 mM Tris, 150 mM NaCl, and 0.1 mM Ethylenediaminetetraacetic acid (EDTA), pH 7.4) as described previously. The leakage of calcein from the LUVs was monitored by measuring fluorescence intensity at an excitation wavelength of 490 nm and an emission wavelength of 520 nm on a model RF-5301PC spectrophotometer (Shimadzu, Kyoto, Japan). For determination of 100% dye-release, 10% Triton-X100 in Tris-buffer (20 μL) was added to dissolve the vesicles. The experiment was performed in triplicate, and the percentage of dye-leakage caused by the peptides was calculated as follows: Dye-leakage (%) = 100 × (F − F_0_)/(F_t_ − F_0_), where F is the fluorescence intensity shown by the peptides and where F_0_ and F_t_ are fluorescence intensities without the peptides and with Triton X-100, respectively.

### 4.10. LAL Assay

The LPS-neutralizing activity of all peptides was determined using the Pierce LAL chromogenic endotoxin quantitation kit (ThermoFisher Scientific, MA, USA). All peptides including standard (Polymixin B) at varying concentrations (3.12, 6.25, 12.5, 25, and 50 μM in endotoxin-free water) were allowed to bind with 20 ng/mL of LPS in a 96-well plate for 30 min at 37 °C. To this, an equal volume (10 μL) of LAL reagent was added and incubated for 10 min at 37 °C and then 20μL of LAL chromogenic substrate was added. After 15 min incubation at 37 °C, the reaction was stopped by 25% acetic acid and the reduction in yellow color formation due to the substrate was measured at 405 nm on an absorbance microplate reader. The experiment was conducted in triplicate, and the changes in absorbance due to peptide-LPS interaction are indicated as percent LPS neutralization.

### 4.11. Scanning Electron Microscope Analysis

Membrane damage of E. coli (KCTC 1682) was visualized by Field Emission Scanning Electron Microscope (FE-SEM) to confirm that the peptides target the bacterial membrane as described previously [62]. The 1× MIC and 2× MIC of peptides were treated to E. coli, diluted in 10 mM PBS buffer to an OD_600_ of 0.2, and incubated for 4 h at 37 °C. The cells were visualized with a FE-SEM (SU8020; Hitachi, Tokyo, Japan) after fixation and dehydration.

### 4.12. Protease Stability Assay

For stability analysis, all Pro-peptides at their MICs were incubated with trypsin and chymotrypsin at the ratio of 10,000:1 (peptide–enzyme) in PBS for 6 h at 37 °C. After incubation, 100 μL of peptide-protease mixture was added to the 100 μL bacterial suspension (*E. coli* and *A. baumannii*, 2 × 10^5^ CFU/mL) and further incubated for 16 h at 37 °C. Melittin was used as standard peptide, and bacterial suspension without peptide and protease served as control. The experiment was repeated three times, and the extent of bacterial growth was measured at 600 nm using a microplate reader.

### 4.13. Biofilm Assay

Pro-peptides were evaluated for their antibiofilm activity against *E. coli, A. baumannii* and MDR strains (MDREC1299 and MDRAB 12010) in vitro. Bacterial suspensions (2 × 10^5^ CFU/mL) were grown in Mueller-Hinton (MH) media supplemented with 0.2% glucose in a 96-well plate and then incubated with Pro-peptides and melittin standard (0, 1, 2, 4, 8, 16, 32, and 64 μM) for 16 h at 37 °C. Supernatants were discarded after incubation, and plates were fixed with 100% methanol for 15 min and then stained with 0.1% (*w*/*v*) crystal violet in 0.25% (*v*/*v*) acetic acid for 1 h. Unbound dye was washed with excess distilled H_2_O and allowed to dry. Plates were dissolved in 95% (*v*/*v*) ethanol, and the degree of biofilm mass was quantified by measuring OD at 595 nm using a microplate reader. The experiments were conducted in triplicate, and the antibiofilm activity was represented as the percentage of biofilm formed with respect to the untreated control.

For microscopic analysis, MDREC 1299 was grown in MH media containing 0.2% glucose on the polystyrene 6-well plate and incubated with Pro10-1D (0–16 μM) for 16 h at 37 °C. After treatment, the contents were carefully discarded, fixed with 100% methanol (15 min), and then stained with 0.4% (*w*/*v*) crystal violet for 1 h at room temperature. The stained plates were examined for inhibition of biofilm formation under light microscope (Eclipse Ni; Nikon, Tokyo, Japan).

### 4.14. Quantification of Nitrite and Inflammatory Cytokine Production in LPS-Stimulated RAW264.7 Cells

The presence of nitrite in culture media strongly validates the indication of NO production which was assessed by Griess assay. Briefly, RAW264.7 cell (1 × 10^5^) were seeded in s 96-well plate in triplicate. Peptides (1, 5, 25, and 50 μM) were pretreated 1 h and then stimulated with 20 ng/mL of LPS purified from *E. coli* O111:B4 (Sigma-Aldrich, St. Louis, MO, USA) for 16 h. After incubation, equal volumes of culture media and Griess reagent were added and color change was read at 540 nm. The concentration of nitrite content was assessed using a standard curve of sodium nitrite. In addition, release of inflammatory cytokines including TNF-α and IL-6 in the culture media was quantified using an enzyme-linked immunosorbent assays kit (ELISA; R&D Systems, Minneapolis, MN, USA) and the assay was performed according to the kit protocol. The assay was conducted in triplicate, and the concentrations of TNF-α and IL-6 were evaluated by measuring the absorbance at 450 nm using a microplate reader.

### 4.15. Western Blot Analysis

The protein content of all experimental groups of RAW264.7 cells in triplicate were extracted using radio immunoprecipitation buffer (RIPA) containing protease phosphatase inhibitor. The supernatant was collected by centrifugation (12,000× g) at 4 °C for 20 min and stored at −80 °C until further use. Similarly, cytoplasmic and nuclear fractionation was extracted using an NE-PER Nuclear and Cytoplasmic Extraction Reagents kit (ThermoFisher Scientific, MA, USA), according to the manufacturer’s protocol. Protein contents were quantified using a bicinchoninic acid (BCA) protein assay kit (Thermo Fisher Scientific Inc., MA, USA) and 30 μg (*v/v*) was electrophoresed by SDS-PAGE using a 10% (*w/v*) polyacrylamide resolving gel, electro-transferred to a PVDF membrane, and then blocked using 5% (*w/v*) non-fat milk. The membranes were incubated with primary antibodies (1:1000 dilution) at 4 °C overnight, followed by incubation with their respective secondary antibody for 1 h at room temperature. The antigen-antibody complexes were examined using the WestGlow^TM^ Chemiluminescent Substrate (Biomax Co., Ltd, Seoul, South Korea) under iBright™ CL1000 Imaging System (Thermo Fisher Scientific Inc., MA, USA). The densitometric analysis of protein signals were quantified using the Image J software (Version 1.52, National Institutes of Health, MD, USA).

### 4.16. Animal

Female Institute of Cancer Research (ICR) mice (4-week-old, 24–25 g) were purchased from Orient (Daejeon, Korea). All mice were housed under specific pathogen-free conditions in a temperature-and humidity-controlled environment. All procedures were approved by the Institutional Animal Care and Use Committee (IACUC) of Konkuk University, Seoul, South Korea (IACUC number: KU19197-1).

### 4.17. Cytotoxicity In Vivo

A preliminary toxicity study was conducted to determine the cytotoxic effect of Pro10-1 and Pro10-1D on ICR mice. Four-week-old mice (*n* = 5) were injected intraperitoneally with peptides (1 mg/Kg and 5 mg/Kg, respectively) for 24 h. After treatment, blood was withdrawn and serum was collected and then subjected to AST, ALT, and BUN analyses [78].

### 4.18. Measurement of Antiseptic Activity of Pro10-1D in E. coli K1 Septic Shock Mouse Model

For sepsis model, we used *E. coli* K1 as an infection strain [47], and mice were randomly sorted in to 6 groups (5 mice/group). Control mice received I.P. injection of phosphate buffered saline (PBS, pH = 7.4) whereas Pro10-1 and Pro10-1D were injected with 1 mg/Kg and served as a peptide control. Mice infected with *E. coli* K1 (6 × 10^6^ CFU/mouse, I.P.) served as a sepsis control. For treatment measures, mice were pretreated with 1 mg/kg of the respective peptides for 1 h and then infected with *E. coli* K1 like the septic mice. After 16 h, mice were euthanized by cervical dislocation under overdose of ether anesthesia. The blood samples, lung, liver, and kidney tissues were excised and processed for biochemical and histological analysis. Briefly, organ lysates of lung, liver, and kidney harvested from ice-cold PBS were evaluated for number of bacterial colonies. Endotoxin levels in the serum contents were quantified according to the kit protocol using Pierce LAL Chromogenic Endotoxin Quantitation Kit (Thermo Fisher Scientific, MA, USA). The levels of AST, ALT, and BUN in serum and the inflammatory cytokine levels in the serum or lung lysates were quantified as described previously [63].

### 4.19. Polymorphonuclear Lymphocyte (PMN) Infiltration

Lung tissues of the control and an experimental group of mice fixed by paraformaldehyde (4% *v*/*v*) were evaluated for histological changes. Briefly, 6-mm-thick lung tissue sections were deparaffinized in xylene, rehydrated with graded ethanol, and then stained with H&E stain. The stained section was examined for neutrophil infiltration and other morbid changes with a light microscope (Eclipse Ni; Nikon, Tokyo, Japan).

### 4.20. Statistical Analysis

All statistical analyses were performed using GraphPad Prism 8 Software (GraphPad Software Inc., La Jolla, CA, USA). The data are presented as the mean ± SEM of three independent experiments. The statistical analysis for hemolysis and dye leakage was analyzed by a nonlinear regression curve fit. For other biochemical and protein expressions, we performed *one-way ANOVA* followed by *Tukey’s honestly significant difference (HSD) post hoc test*. Values indicate statistically significant at * *p* < 0.05, ** *p* < 0.01, and *** *p* < 0.001; ns represents nonsignificant.

## 5. Conclusions

Our study reveals the importance of a newly designed 10-mer peptide, Pro10-1D, and its systemic efficacy against gram-negative pathogens. Among the analog peptides, the addition of Arg and D-amino acid conversion in Pro10-1D increased cationicity and reduced hydrophobicity, resulting in enhanced permeabilization of the negatively charged bacterial membrane. Pro10-1D showed excellent antibacterial activities, resistance to proteolytic digestion, endotoxin clearance, and antibiofilm activities, which are essential features of potent peptide antibiotics. Pro10-1D targets gram-negative bacterial strains through membrane disruption. Furthermore, Pro10-1D exerts substantial anti-inflammatory effects at subinhibitory doses by suppressing LPS-stimulated cascades downstream of TLR4 in vitro. Finally, experiments in the *E. coli* K1-induced septic mouse model confirmed that Pro10-1D undoubtedly ameliorates the overwhelming inflammatory insults and shows protective effects against organ dysfunction in mice with sepsis. Therefore, the development of Pro10-1D as a therapeutic against gram-negative bacteria through modulation of the parent peptide validates the potential of developing novel peptide antibiotics for the effective treatment of gram-negative pathogen-induced sepsis.

## Figures and Tables

**Table 1:** Peptides and their physicochemical properties.

Peptides	Sequence ^a^	Length	Molecular Weight	Charge	Hydrophobic Moment <µH> ^b^	Hydrophobicity <H> ^b^
Pro9-3	RLWLAIWRR-NH2	9	1269	+3	0.692	0.776
Pro9-3D	rlwlaiwrr-NH2	9	1269	+3	0.692	0.776
Pro10-1	RRLWLAIWRR-NH2	10	1424	+4	0.584	0.597
Pro10-1D	rrlwlaiwrr-NH2	10	1424	+4	0.584	0.597

^a^ Small letters in sequence represents D-amino acids. ^b^ Hydrophobic moment <µH> and hydrophobicity <H> were calculated by HeliQuest online tool [32].

**Table 2:** Activities of Pro9-3 and its analogs against microorganisms.

Microorganisms		Minimal Inhibitory Concentration (MIC_100_) in μM			
	Pro9-3	Pro9-3D	Pro10-1	Pro10-1D	Melittin
Standard gram-negative bacteria	16	8	16	8	8
*E. Coli*	16	4	16	4	8
*E. coli K1*					
*A. baumannii*	16	4	8	2	4
MDR Gram-negative bacteria					
MDREC 1229	16	8	8	4	16
MDREC 1238	32	4	32	4	32
MDRAB 12010	16	8	8	4	8
MDRAB 12220	16	8	16	8	8
^a^ GM	18.29	6.29	14.86	4.86	12.0
^b^ HC_10_	200	200	200	200	0.8
^c^ Relative selective index	10.94	31.82	13.46	41.18	0.07

^a^ The geometric means (GMs) are the mean minimum inhibitory concentration (MIC) values of all bacterial strains. The average MIC was calculated from three experimental measurements. ^b^ HC_10_ is the degree of peptide concentration inducing 10% hemolysis of heparinized sheep red blood cells in vitro. When no detectable hemolysis was observed at 100 μM, 200 μM was used to calculate the relative selective index. ^c^ Relative selective index was calculated using GM of the MIC (μM)/HC_10_. The larger values indicate greater cell selectivity.
